# Facility and Geographic Variation in Rates of Successful Community Discharge After Inpatient Rehabilitation Among Medicare Fee-for-Service Beneficiaries

**DOI:** 10.1001/jamanetworkopen.2018.4332

**Published:** 2018-11-09

**Authors:** Addie Middleton, James E. Graham, Janet Prvu Bettger, Allen Haas, Kenneth J. Ottenbacher

**Affiliations:** 1 Division of Physical Therapy, Medical University of South Carolina, Charleston; 2 Department of Occupational Therapy, Colorado State University, Fort Collins; 3 Department of Orthopedic Surgery, Duke Clinical Research Institute, Duke University, Durham, North Carolina; 4 Department of Preventative Medicine and Community Health, The University of Texas Medical Branch, Galveston; 5 Division of Rehabilitation Sciences, The University of Texas Medical Branch, Galveston

## Abstract

**Importance:**

The Improving Medicare Post–Acute Care Transformation (IMPACT) Act of 2014 mandated a quality measure of successful community discharge for postacute care services. Examining variation in performance nationally can help identify opportunities for improving patient-centered quality of care.

**Objective:**

To examine US facility-level and geographic variation in rates of successful community discharges after inpatient rehabilitation.

**Design, Setting, and Participants:**

This retrospective cohort study of Medicare claims data from December 31, 2013, through October 1, 2015, included 1154 inpatient rehabilitation facilities submitting claims to the Centers for Medicare & Medicaid Services and a total of 487 862 Medicare fee-for-service beneficiaries discharged from inpatient rehabilitation facilities. Analyses were performed from December 8, 2017, through September 11, 2018.

**Main Outcomes and Measures:**

Successful community discharge as defined for the Discharge to Community—Post–Acute Care Inpatient Rehabilitation Facility Quality Reporting Program measure. To be considered a successful community discharge, patients had to discharge from the inpatient rehabilitation facility to the community (ie, home or self-care) and remain there without experiencing an unplanned rehospitalization or dying within the following 31 days. Centers for Medicare & Medicaid Services specifications were followed to identify the cohort, define the outcome, and calculate risk-standardized facility and state rates.

**Results:**

Among the 487 862 patients included in the cohort, mean (SD) age was 76.4 (10.8) years, and 56.9% were female. The overall rate of successful community discharge after inpatient rehabilitation was 63.7% (95% CI, 63.6%-63.8%). Risk-standardized rates ranged from 42.9% to 83.6% across inpatient rehabilitation facilities. Two hundred sixteen facilities (18.7%) performed significantly better than the mean national rate and 203 (17.6%) performed significantly worse (*P* < .05). Risk-standardized state rates ranged from 55.9% to 73.3%. Rates were lowest in the Northeast (Massachusetts, 55.9%; New Hampshire, 57.0%) and highest in the West (Oregon, 70.3%; Hawaii, 73.3%).

**Conclusions and Relevance:**

The observed variation suggests opportunities exist for improving this important, patient-centered national quality measure. Future research is needed to identify the aspects of care delivery and the community services and supports that facilitate successful community discharge. These findings can be used to guide care improvement efforts and further improve the consistency and quality of postacute care.

## Key Points

**Question:**

Do rates of successful community discharge after inpatient rehabilitation vary across US facilities and geographic regions?

**Findings:**

In this cohort study of 487 862 Medicare fee-for-service beneficiaries discharged from 1154 inpatient rehabilitation facilities submitting claims, risk-standardized rates of successful community discharge ranged from 42.9% to 83.6%. Rates were lowest in the Northeast (Massachusetts, 55.9%; New Hampshire, 57.0%) and highest in the West (Oregon, 70.3%; Hawaii, 73.3%).

**Meaning:**

The observed facility and geographic variations suggest opportunities for improving this important, patient-centered, and nationally reported quality outcome.

## Teaser

This cohort study examines facility-level and geographic variation in rates of successful community discharges after inpatient rehabilitation among fee-for-service Medicare beneficiaries.

## Introduction

The introduction of value-based payment has resulted in profound changes in the delivery of health care in the United States,^1^ including the development of quality reporting programs and patient-centered quality measures.^2^ Postacute care has been the focus of recent value-based payment initiatives for several reasons. Medicare fee-for-service spending for postacute care has doubled since 2001 and totaled $60 billion in 2016.^3^ Discharge to postacute care services has increased nearly 50% during the past 15 years, and 42% of Medicare beneficiaries are now discharged from acute care hospitals to postacute care.^2,4^ A report by the National Academy of Sciences found that postacute care services are responsible for the largest geographic variation in Medicare costs when compared with acute care and outpatient services.^5^ Thus, postacute care services represent an important opportunity to improve quality and reduce costs.^5,6^

Substantial research has been conducted examining the role of facility characteristics and geographic location on variation in patient outcomes and health care costs.^7,8,9^ Most of this research has focused on acute care hospitals and outpatient services. Less research has focused on postacute care, in particular for inpatient rehabilitation facilities.

Inpatient rehabilitation facilities provide comprehensive and intensive postacute medical and rehabilitative services.^4^ The goal is to prepare individuals for the most independent living setting possible by facilitating recovery, addressing adaptive equipment needs, and educating patients and their caregivers. Ideally, these additional services allow the individual to discharge back to the community, rather than remain in institutional care.^4^

Successful community discharge is one of the standardized outcome measures specified by the Improving Medicare Post–Acute Care Transformation (IMPACT) Act of 2014 and will be publicly reported for inpatient rehabilitation facilities beginning in 2018.^10,11^ Successful discharge to the community after inpatient rehabilitation is important to the full spectrum of stakeholders, from patients to policy makers. A first step toward improving community discharge rates is to better understand variations in performance, because variation suggests room for improvement. The purpose of our study was to examine facility-level and geographic variation in rates of successful community discharge after inpatient rehabilitation. Findings will help guide the next steps in care improvement initiatives targeting successful community discharge and reducing the cost of institutional care.

## Methods

### Data Sources

Analyses and reporting for this study were conducted following the Strengthening the Reporting of Observational Studies in Epidemiology (STROBE) reporting guideline for cohort studies.^12^ We used the following 100% national Medicare files: Medicare Provider Analysis and Review (MedPAR), Inpatient Rehabilitation Facility–Patient Assessment Instrument (IRF-PAI), Beneficiary Summary, and Provider of Service. The MedPAR files contain finalized claims for stays in acute care hospitals, inpatient rehabilitation facilities, psychiatric hospitals, and skilled nursing facilities. We used these files to gather information on patients’ prior hospitalizations, verify inpatient rehabilitation stays (ie, matching admission and discharge dates in IRF-PAI ± 1 day), and identify rehospitalizations within the 31-day window after inpatient rehabilitation discharge. The IRF-PAI files were used to extract information on patients’ inpatient rehabilitation stay, including their initial discharge destination. Beneficiary Summary files were used to gather sociodemographic and Medicare enrollment information and to identify patients who died within the 31-day window after inpatient rehabilitation. Files were linked using unique, encrypted patient identifiers. All analyses were completed after establishing a data use agreement with the Centers for Medicare & Medicaid Services (CMS) and obtaining approval from the institutional review board of The University of Texas Medical Branch, Galveston, which waived the need for informed consent for use of deidentified data from publicly available files.

### Patient Population

**Figure 1.**
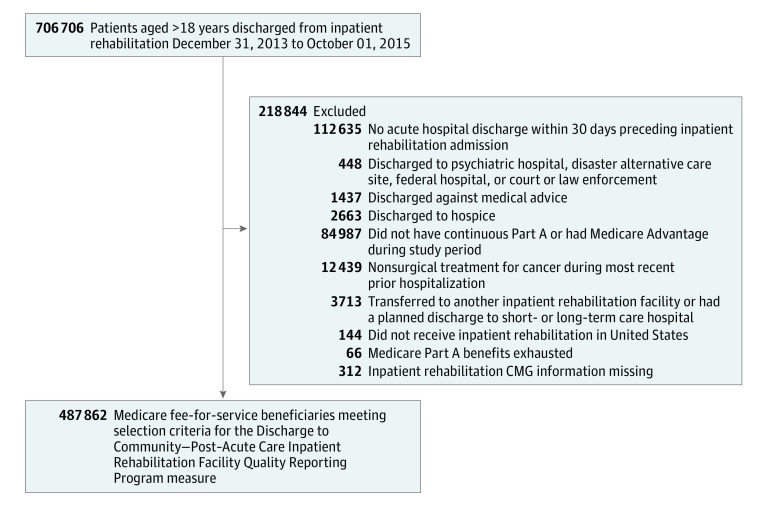
Cohort Selection The study period refers to the 12 months before inpatient rehabilitation through 31 days after discharge for each stay. CMG indicates case-mix group.

The final cohort included 487 862 Medicare fee-for-service beneficiaries discharged from inpatient rehabilitation from December 31, 2013, through October 1, 2015 (Figure 1). We identified our cohort using the exclusion criteria for the Discharge to Community—Post–Acute Care Inpatient Rehabilitation Facility Quality Reporting Program (Community Discharge IRF-QRP) measure.^13^ Individuals younger than 18 years and with no acute care stay during the 30 days before inpatient rehabilitation admission were excluded. We also excluded those discharged from inpatient rehabilitation to psychiatric hospitals, disaster alternative care sites, federal hospitals, and court or law enforcement, as well as patients who discharged against medical advice or to hospice. The risk adjustors and outcome for the Community Discharge IRF-QRP measure require a 1-year look back before inpatient rehabilitation and a 31-day observation period after discharge. Therefore, patients were excluded if they were not continuously enrolled in Medicare fee-for-service during that time. We also excluded patients whose prior hospitalization was for nonsurgical treatment of cancer, those transferred to another inpatient rehabilitation facility or with a planned discharge to a short- or long-term care hospital, patients receiving care outside of the United States or US territory, patients who exhausted their Medicare Part A benefits during their inpatient rehabilitation stay, and patients with missing inpatient rehabilitation case-mix group (CMG) information.^13^

### Outcome

The outcome was successful community discharge as defined for the Community Discharge IRF-QRP measure.^13^ The intent of CMS’s new quality outcome measure is to capture successful discharges to the community. To be considered a successful community discharge, patients must discharge from the inpatient rehabilitation facility to the community (ie, home or self-care) and remain there without experiencing an unplanned rehospitalization or dying within the next 31 days.^13^ We used the discharge destination codes specified for the quality measure to identify community discharges, and then reviewed hospital claims and beneficiary death dates to determine success.

### Statistical Analysis

Data were analyzed from December 8, 2017, through September 11, 2018. We calculated risk-standardized rates of successful community discharge for all inpatient rehabilitation facilities submitting claims to CMS. Risk-standardized rates are used for quality reporting and are the ratio of a facility’s predicted number of successful community discharges to their expected number, multiplied by the mean rate across all facilities. We used hierarchical logistic regression to calculate the facilities’ predicted and expected numbers of community discharges, where the predicted number included the random intercept and the expected number did not. We replicated as closely as possible the risk adjustors specified for the Community Discharge IRF-QRP measure,^13^ which included patients’ age and sex groups, original reason for Medicare entitlement (ie, age, end-stage renal disease, or disability), number of acute care stays during the previous year (count), primary diagnosis and/or surgical category from the prior acute stay, receipt of dialysis during the prior acute stay (yes/no), length of prior acute stay (in days [categorical]) or prior stay in a psychiatric hospital, comorbidities, and inpatient rehabilitation CMG. Inpatient rehabilitation CMGs are used by CMS to determine payment under the inpatient rehabilitation prospective payment system. The CMGs are based on patients’ impairment category (eg, stroke, lower extremity fracture), functional status (ie, motor and cognition), age, and comorbidity tier.^14^ Primary diagnoses and surgical procedures (*International Classification of Diseases, Ninth Revision* [*ICD-9*] codes) from patients’ prior acute care stays were categorized into the Clinical Classifications Software groups developed by the Agency for Healthcare Research and Quality. We categorized comorbidities into the Hierarchical Condition Categories used by CMS. The Hierarchical Condition Categories were identified by reviewing secondary diagnoses (*ICD-9* codes) from the most recent hospitalization or all hospitalizations during the prior year based on CMS specifications.^15^ The Clinical Classifications Software groupings and Hierarchical Condition Categories are the classification approaches used for primary hospital diagnoses and comorbidities in risk adjustment for the Community Discharge IRF-QRP measure.^13^

We used bootstrapping to calculate 95% CI estimates for facility-level risk-standardized rates.^13^ These CIs were used to identify inpatient rehabilitation facilities performing significantly better and significantly worse than the mean national rate. To describe facilities performing significantly better and worse than the mean national rate, we examined the following characteristics: size (bed count), ownership (government, nonprofit, or for-profit), teaching status (teaching or nonteaching), and location (urban or rural). Facility characteristics were extracted from CMS Provider of Service files.^16^ We also examined facility-level mean motor and cognitive functional scores at discharge. The risk-standardized rates used to identify facilities performing significantly better and worse than the mean on the community discharge quality measure are adjusted for patients’ CMG, which includes admission functional status. Examining discharge scores provides insight into functional outcomes among facilities with higher and lower risk-adjusted successful community discharge rates. Functional status data were extracted from IRF-PAI files, which include items from the Functional Independence Measure.^17^ These items are rated on a 7-point scale, with higher scores indicating greater functional independence. Motor subscale scores were calculated from 13 items related to self-care, sphincter control, mobility, and locomotion. Cognition subscale scores were calculated from 5 items related to comprehension, expression, social interaction, and memory. We categorized motor and cognition scores into quartiles and examined distributions across the score quartiles of inpatient rehabilitation facilities performing significantly better and worse than the mean national rate on the successful community discharge quality measure.

To examine geographic variation, we calculated risk-standardized state rates of successful community discharge after inpatient rehabilitation. We used the same risk adjustors that were included in the facility-level risk standardized rates. We used bootstrapping to calculate 95% CI estimates for the risk-standardized state rates. All analyses were performed using SAS (version 9.4; SAS Institute Inc) and SPSS (version 24; IBM Corporation) software. *P* < .05 indicated significance using a 2-tailed test.

## Results

**Figure 2.**
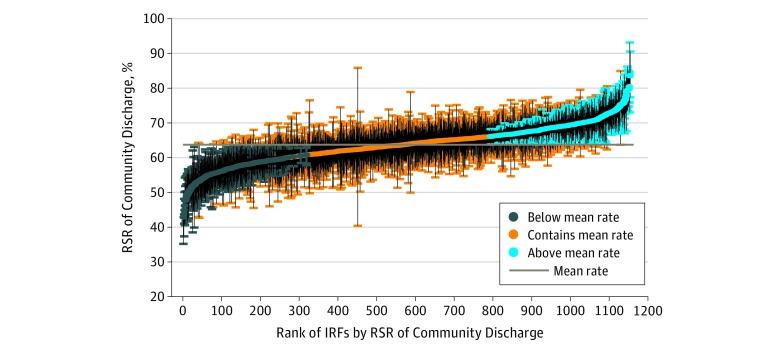
Risk-Standardized Rates (RSRs) of Successful Community Discharge After Inpatient Rehabilitation Rates are shown for the 1154 inpatient rehabilitation facilities submitting claims to the Centers for Medicare & Medicaid Services during the study period. Facilities are shown in rank order, with a dot representing their RSR of community discharge and a corresponding vertical line representing the 95% CI for the rate. Two hundred sixteen inpatient rehabilitation facilities (18.7%) had 95% CIs entirely above the overall mean rate; 419 facilities (63.7%), 95% CIs that contain the mean rate; and 203 facilities (17.6%), 95% CIs entirely below the mean rate. IRF indicates inpatient rehabilitation facility.

The mean (SD) age of this cohort of 487 862 Medicare beneficiaries was 76.4 (10.8) years; 277 129 (56.9%) were women and 210 733 (43.2%) were men. The overall rate of successful community discharge after inpatient rehabilitation was 63.7% (95% CI, 63.6%-63.8%). The hierarchical logistic regression model estimating successful community discharge is presented in eTable 1 in the Supplement. Risk-standardized rates ranged from 42.9% to 83.6% across the 1154 inpatient rehabilitation facilities submitting claims to CMS during the study period (Figure 2). Two hundred sixteen facilities (18.7%) performed significantly better than the mean national rate and 203 (17.6%) performed significantly worse (*P* < .05). Characteristics of the facilities performing significantly better and worse than the mean national rate are presented in the Table. Although distributions across characteristics differed between facilities performing significantly better and worse than the mean national rate, consistencies in patterns were observed. Among both performance groups, the largest percentage had bed counts less than 200 (117 of 216 [54.2%] and 79 of 203 [38.9%]), and most were in an urban location (189 of 216 [87.5%] and 185 of 203 [91.1%]). Differences were observed in ownership and teaching status. Most facilities performing worse than the mean national rate were nonprofit (128 of 203 [63.1%]), and most facilities performing better than the mean national rate were nonteaching (151 of 216 [69.9%]).

**Table.** Characteristics of High- and Low-Performing Inpatient Rehabilitation Facilities on the Successful Community Discharge Quality Measure

Facility Characteristic	Performance of Facilities, No. (%)^a^	
	High (n = 216)^b^	Low (n = 203)
Facility total bed count		
0-200	117 (54.2)	79 (38.9)
201-350	36 (16.7)	46 (22.7)
351-500	19 (8.8)	39 (19.2)
>500	41 (19.0)	39 (19.2)
Ownership		
Government	23 (10.6)	26 (12.8)
Not-for-profit	96 (44.4)	128 (63.1)
Profit	94 (43.5)	49 (24.1)
Teaching status		
Nonteaching	151 (69.9)	104 (51.2)
Teaching	62 (28.7)	99 (48.8)
Location		
Rural	24 (11.1)	18 (8.9)
Urban	189 (87.5)	185 (91.1)
Mean discharge Motor score^c^		
Quartile 1 (18.0-58.3)	33 (15.3)	68 (33.5)
Quartile 2 (58.4-61.0)	54 (25.0)	65 (32.0)
Quartile 3 (61.1-63.9)	52 (24.1)	46 (22.7)
Quartile 4 (64.0-74.3)	77 (35.6)	24 (11.8)
Mean discharge Cognitive score^d^		
Quartile 1 (10.0-26.5)	42 (19.4)	55 (27.1)
Quartile 2 (26.6-27.6)	54 (25.0)	46 (22.7)
Quartile 3 (27.7-28.8)	68 (31.5)	57 (28.1)
Quartile 4 (28.9-33.8)	52 (24.1)	45 (22.2)

^a^ Performance refers to inpatient rehabilitation facilities performing significantly better (high) and significantly worse (low) than the mean national rate on the successful community discharge quality measure, respectively.

^b^ Facility characteristic data were missing from Centers for Medicare & Medicaid Services Provider of Service files for 3 inpatient rehabilitation facilities (n = 213). Discharge functional data are complete.

^c^ Measured using the Motor subscale of the Functional Independence Measure. Scores range from 0 to 91 points, with higher scores indicating better motor functional independence.

^d^ Measured using the Cognition subscale of the Functional Independence Measure. Scores range from 0 to 35 points, with higher scores indicating better cognitive independence.

Differences in motor functional outcomes were also observed. Seventy-seven facilities (35.6%) performing significantly better than the mean national rate on the successful community discharge quality measure had mean motor discharge scores in the top quartile compared with only 24 (11.8%) performing significantly worse than the mean national rate. The distribution across cognition score quartiles was fairly similar between facilities performing significantly better and worse than the mean national rate on the successful community discharge quality measure. The largest difference was observed in the lowest cognition score quartile. Fifty-five of 203 facilities (27.1%) performing significantly worse than the mean national rate on the successful community discharge quality measure had mean discharge cognition scores in the lowest quartile compared with 42 of 216 facilities (19.4%) performing significantly better than the mean national rate.

**Figure 3.**
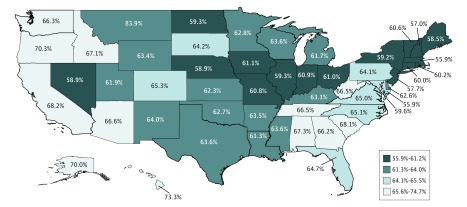
Risk-Standardized State Rates of Successful Community Discharge After Inpatient Rehabilitation States are color coded by performance quartile of successful community discharge rate.

Risk-standardized state rates of successful community discharge after inpatient rehabilitation ranged from 55.9% to 73.3% (Figure 3). Rates were lowest in the Northeast (Massachusetts, 55.9%; New Hampshire, 57.0%) and Midwest (Nebraska, 58.9%; Illinois, 59.3%; North Dakota, 59.3%) and highest in the West (Oregon, 70.3%; Hawaii, 73.3%) and in the Southeast (South Carolina, 68.1%; Alabama, 67.3%). Risk-standardized state rates and corresponding 95% CIs are presented in eTable 2 in the Supplement.

## Discussion

Returning to and remaining in the community after an illness or injury is an important patient-centered outcome reflecting the quality of postacute care.^4,13,18^ Variation has been observed in rates of discharge to the community based on the initial destination after inpatient rehabilitation^13,19^ and in rates of community discharge without rehospitalization during 30 days.^4^ However, a better understanding of variation in successful community discharges, as defined by the new quality metric, is needed. In this national cohort of Medicare fee-for-service beneficiaries, rates of successful community discharge after inpatient rehabilitation varied across facilities and geographic regions. The observed variation suggests that we may be able to improve quality and reduce costs of inpatient postacute care.^20^

Approximately 75% of patients are initially discharged to the community after inpatient rehabilitation.^4,21^ Reported rates range from 68% to 70% for patients with hip fracture,^22,23,24^ 70% to 76% for patients with stroke,^19,25,26,27,28^ 72% for patients with traumatic spinal cord injury,^29^ 74.2% for patients with traumatic brain injury,^30^ and 91.9% for patients with lower-extremity joint replacement.^31^ These rates reflect patients’ planned discharge destination and do not capture whether the individual is able to remain in the community.^19,21,22,23,24,25,26,27,28,29,30,31^ Leland et al^32^ examined successful community discharges in a cohort of patients with hip fracture. They defined success as discharging from postacute care (inpatient rehabilitation or skilled nursing facility) to the community and remaining in the community for 30 days without a subsequent reentry into the health care system or death.^32^ Their findings highlight the need to look beyond patients’ initial discharge setting, because 14% of patients initially discharged to the community did not remain there for 30 days.^32^

Initial work has focused on the patient characteristics associated with successful community discharge after inpatient rehabilitation. Cary et al^33^ examined successful community discharge in a cohort of Medicare beneficiaries discharged from inpatient rehabilitation in 2013. Their cohort and definition of successful community discharge differed from those of our study. Their cohort was restricted to those discharged to noninstitutional settings, and success was defined as surviving and having no acute or postacute admission during the 30 days after inpatient rehabilitation discharge. Despite these differences, the findings provide insight regarding the patient characteristics associated with better community discharge outcomes after inpatient rehabilitation. In their cohort, younger age, male sex, social support, fewer comorbid conditions, better functional status at inpatient rehabilitation admission, and less use of hospital services during the prior year were associated with higher odds of successful community discharge.^33^ These findings indicate that sociodemographic and clinical characteristics are associated with successful community discharge, and our findings suggest that room for improvement exists at the facility level.

Our findings provide initial insight into characteristics of facilities performing significantly better and significantly worse than the mean national rate on the successful community discharge quality measure. However, analyses of facility characteristics were limited to bed count, ownership, teaching status, and urban or rural location. Achieving better functional outcomes may contribute to higher community discharge rates, because we observed better motor functional outcomes among higher performing facilities. Other modifiable aspects of care delivery likely promote successful community discharge. Future studies should examine the processes and programs in inpatient rehabilitation facilities with high- vs low-risk standardized rates of successful community discharge.

Another avenue for future research is to better understand regional differences in rates of successful community discharge. Regional differences in community discharge rates have been previously reported among patients with stroke.^19^ That study focused solely on patients with stroke, and the outcome was community as the initial setting, rather than successful community discharge as defined by the inpatient rehabilitation quality metric. Despite these differences, our findings are consistent with those of the prior study, which also reported lower rates in the Northeast and higher rates in the West.^19^ The consistency of these patterns supports the need for future research to understand regional differences in care processes and/or community services and supports.

### Broader Implications

Successful community discharge is a new quality measure mandated by the IMPACT Act of 2014.^10^ The measure is standardized across all postacute care settings, which include inpatient rehabilitation facilities, skilled nursing facilities, long-term care hospitals, and home health care agencies.^10^ A concern with newly implemented quality metrics is the potential for unintended consequences. The characteristics of individuals receiving postacute care will need to be monitored as the community discharge quality metric is implemented to ensure that disparities in access do not emerge. The intended consequence of the new quality measure is to incentivize health care providers to improve processes that influence patients’ ability to successfully return to community settings. However, not all patients will be appropriate for discharge to the community. The new measure does not penalize postacute care providers for institutionalizations (eg, long-term care nursing home admissions) occurring after an initial discharge to a community setting. Monitoring rates of institutionalization after discharge to the community will provide insight into whether inappropriate community discharges may be an unintended consequence of the new quality measure.

Another concern with new quality measures is whether the metric, as defined, truly reflects quality of care. Future research is needed to validate the successful community discharge measure as an indicator of high-quality care. The premise of the measure as a quality indicator is twofold. Community discharge is an important outcome for patients and families, and discharge to community settings is associated with lower health care costs than discharge to institutional settings.^13^ For these reasons the successful community discharge measure will be used by CMS to assess quality of postacute care.

Tracking patient-centered quality measures, such as community discharge rates, will be imperative as postacute care reforms are implemented. The Medicare Payment Advisory Commission continues to refine recommendations for a unified postacute care prospective payment system.^3^ Currently, postacute health care providers are reimbursed by Medicare using setting-specific payment systems.^3^ Under a unified payment system, reimbursement for services will be based on patient characteristics and outcomes rather than the postacute care setting.^3^ To fully understand the effects of a unified payment system, variation in patient outcomes within and across postacute care settings will need to be monitored during and after the payment reform.

Successful community discharge rates after postacute care also have implications in the context of episode-based payment models. Episode-based payment models incentivize the accountable entity to improve outcomes and minimize costs throughout the episode of care.^34^ Currently, costs for postacute care services vary across settings,^2^ which may influence decisions on the type of postacute care patients receive under episode-based payment models. Reporting of successful community discharge rates will allow acute health care providers to identify high-quality postacute health care providers to partner with in episode-based payments.

In their June 2018 Report to Congress, the Medicare Payment Advisory Commission highlighted the importance of beneficiaries receiving postacute care from high-quality health care providers.^3^ The premise is that high-quality postacute care leads to better patient outcomes and lower downstream health care spending.^3^ Our findings suggest that opportunities exist for further improving the quality of postacute care. The next step is identifying the aspects of care delivery and community services and supports that facilitate successful community discharge. Continuing to improve the quality of postacute care is critical as we shift to a health care payment system that rewards value.^1,10^

### Limitations

We used the specifications for the Community Discharge IRF-QRP measure to identify our cohort and calculate risk-standardized rates. These rates are not adjusted for patients’ race/ethnicity or Medicaid eligibility, which are social determinants that may have an effect on successful community discharge.^32^ Some of the variation observed may be due to unmeasured confounding. The definition of success does not take into account admission to institutional settings other than acute care hospitals within the 31 days after inpatient rehabilitation. This definition may not align with patient and caregiver perceptions of success; however, our intent was to replicate the quality measure.

## Conclusions

In this study, risk-standardized rates of successful community discharge ranged from 42.9% to 83.6% across inpatient rehabilitation facilities and from 55.9% to 73.3% across states. The facility and regional variation observed suggests there may be opportunities for improving this important, patient-centered quality measure. Future research is needed to identify the aspects of care delivery and the community services and supports that facilitate successful community discharge. These findings can be used to guide care improvement efforts and further enhance the quality of postacute care while reducing institutional costs.

## Notes

Supplement. **eTable 1.** Hierarchical Logistic Regression Model Estimating Successful Discharge to the Community After Inpatient Rehabilitation **eTable 2. **Risk-Standardized State Rates of Successful Community Discharge After Inpatient Rehabilitation and Corresponding 95% Confidence Intervals

## References

[1] Burwell SM Setting value-based payment goals: HHS efforts to improve US health care. N Engl J Med. 2015;372(10):-. doi:10.1056/NEJMp1500445 25622024

[2] Medicare Payment Advisory Commission Report to the Congress: Medicare and the health care delivery system. http://www.medpac.gov/docs/default-source/reports/june-2016-report-to-the-congress-medicare-and-the-health-care-delivery-system.pdf?sfvrsn=0. June 2016. Accessed July 3, 2018.

[3] Medicare Payment Advisory Commission Report to the Congress: Medicare and the health care delivery system. http://www.medpac.gov/docs/default-source/reports/jun18_medpacreporttocongress_sec.pdf?sfvrsn=0. June 2018. Accessed July 2, 2018.

[4] Medicare Payment Advisory Commission Report to the Congress: Medicare payment policy. http://medpac.gov/docs/default-source/reports/mar17_entirereport224610adfa9c665e80adff00009edf9c.pdf?sfvrsn=0. March 2017. Accessed July 4, 2018.

[5] Institute of Medicine Variation in Health Care Spending: Target Decision Making, Not Geography. Washington, DC: National Academy of Sciences; 2013. 24851301

[6] Newhouse JP, Garber AM Geographic variation in health care spending in the United States: insights from an Institute of Medicine report. JAMA. 2013;310(12):1227-1228. doi:10.1001/jama.2013.278139 24008265

[7] Hussey PS, Huckfeldt P, Hirshman S, Mehrotra A Hospital and regional variation in Medicare payment for inpatient episodes of care. JAMA Intern Med. 2015;175(6):1056-1057. doi:10.1001/jamainternmed.2015.0674 25867180

[8] Medicare Payment Advisory Commission Report to the Congress: regional variation in Medicare service use. http://www.medpac.gov/docs/default-source/reports/Jan11_RegionalVariation_report.pdf?sfvrsn=0. January 2011. Accessed July 9, 2018.

[9] Zhang Y, Baik SH, Fendrick AM, Baicker K Comparing local and regional variation in health care spending. N Engl J Med. 2012;367(18):1724-1731. doi:10.1056/NEJMsa1203980 23113483 PMC3490218

[10] Improving Medicare Post–Acute Care Transformation (IMPACT) Act of 2014. PL 113-185, 128 Stat 1952. https://www.govtrack.us/congress/bills/113/hr4994. Accessed July 4, 2018.

[11] Department of Health and Human Services, Centers for Medicare & Medicaid Services. 42 CFR Part 412. Medicare Program; Inpatient Rehabilitation Facility Prospective Payment System for Federal Fiscal Year 2018 https://www.gpo.gov/fdsys/pkg/FR-2017-08-03/pdf/2017-16291.pdf. Accessed July 4, 2018.

[12] Equator Network. The Strengthening the Reporting of Observational Studies in Epidemiology (STROBE) Statement: Guidelines for Reporting Observational Studies. http://www.equator-network.org/reporting-guidelines/strobe/. Updated July 30, 2018. Accessed August 30, 2018.

[13] RTI International; Center for Clinical Standards and Quality. Measure Specifications for Measures Adopted in the FY 2017 IRF QRP Final Rule. https://www.cms.gov/Medicare/Quality-Initiatives-Patient-Assessment-Instruments/IRF-Quality-Reporting/Downloads/Measure-Specifications-for-FY17-IRF-QRP-Final-Rule.pdf. July 2016. Accessed July 4, 2018.

[14] Department of Health and Human Services; Centers for Medicare & Medicaid Services. Instructions for Implementing the Inpatient Rehabilitation Facility Prospective Payment System (IRF PPS). Program Memorandum Intermediaries. Transmittal A-01-92. https://www.cms.gov/Regulations-and-Guidance/Guidance/Transmittals/downloads/A0192.pdf. July 31, 2001. Accessed September 4, 2018.

[15] Centers for Medicare & Medicaid Services 2014 Model Software/ICD-9-CM Mappings. Version 22 CMS-HCC Risk-Adjustment Model. https://www.cms.gov/Medicare/Health-Plans/MedicareAdvtgSpecRateStats/Risk-Adjustors.html. Modified July 31, 2018. Accessed July 4, 2018.

[16] Centers for Medicare & Medicaid Services Provider of Services Current Files (POS). https://www.cms.gov/Research-Statistics-Data-and-Systems/Downloadable-Public-Use-Files/Provider-of-Services/. Modified July 16, 2018. Accessed September 4, 2018.

[17] Centers for Medicare & Medicaid Services The Inpatient Rehabilitation Facility–Patient Assessment Instrument (IRF-PAI) Training Manual. https://www.cms.gov/Medicare/Medicare-Fee-for-Service-Payment/InpatientRehabFacPPS/IRFPAI.html. Modified July 31, 2018. Accessed June 16, 2016.

[18] Gregory P, Edwards L, Faurot K, Williams SW, Felix AC Patient preferences for stroke rehabilitation. Top Stroke Rehabil. 2010;17(5):394-400. doi:10.1310/tsr1705-394 21131265

[19] Reistetter TA, Karmarkar AM, Graham JE, Regional variation in stroke rehabilitation outcomes. Arch Phys Med Rehabil. 2014;95(1):29-38. doi:10.1016/j.apmr.2013.07.018 23921200 PMC4006274

[20] Chandra A, Dalton MA, Holmes J Large increases in spending on postacute care in Medicare point to the potential for cost savings in these settings. Health Aff (Millwood). 2013;32(5):864-872. doi:10.1377/hlthaff.2012.1262 23650319 PMC3675656

[21] Graham JE, Prvu Bettger J, Middleton A, Spratt H, Sharma G, Ottenbacher KJ Effects of acute-postacute continuity on community discharge and 30-day rehospitalization following inpatient rehabilitation. Health Serv Res. 2017;52(5):1631-1646. doi:10.1111/1475-6773.12678 28580725 PMC5583304

[22] Cary MP, Baernholdt M, Anderson RA, Merwin EI Performance-based outcomes of inpatient rehabilitation facilities treating hip fracture patients in the United States. Arch Phys Med Rehabil. 2015;96(5):790-798. doi:10.1016/j.apmr.2015.01.003 25596000 PMC4410059

[23] Granger CV, Reistetter TA, Graham JE, The Uniform Data System for Medical Rehabilitation: report of patients with hip fracture discharged from comprehensive medical programs in 2000-2007. Am J Phys Med Rehabil. 2011;90(3):177-189. doi:10.1097/PHM.0b013e31820b18d7 21297397 PMC3147279

[24] Wang CY, Graham JE, Karmarkar AM, Reistetter TA, Protas EJ, Ottenbacher KJ FIM motor scores for classifying community discharge after inpatient rehabilitation for hip fracture. PM R. 2014;6(6):493-497. doi:10.1016/j.pmrj.2013.12.008 24389348 PMC4065818

[25] Granger CV, Markello SJ, Graham JE, Deutsch A, Ottenbacher KJ The Uniform Data System for Medical Rehabilitation: report of patients with stroke discharged from comprehensive medical programs in 2000-2007. Am J Phys Med Rehabil. 2009;88(12):961-972. doi:10.1097/PHM.0b013e3181c1ec38 19935180

[26] Ouellette DS, Timple C, Kaplan SE, Rosenberg SS, Rosario ER Predicting discharge destination with admission outcome scores in stroke patients. NeuroRehabilitation. 2015;37(2):173-179. doi:10.3233/NRE-151250 26484509

[27] Reistetter TA, Graham JE, Deutsch A, Granger CV, Markello S, Ottenbacher KJ Utility of functional status for classifying community versus institutional discharges after inpatient rehabilitation for stroke. Arch Phys Med Rehabil. 2010;91(3):345-350. doi:10.1016/j.apmr.2009.11.010 20298822 PMC2896793

[28] Dobrez D, Heinemann AW, Deutsch A, Manheim L, Mallinson T Impact of Medicare’s prospective payment system for inpatient rehabilitation facilities on stroke patient outcomes. Am J Phys Med Rehabil. 2010;89(3):198-204. doi:10.1097/PHM.0b013e3181c9fb40 20068431

[29] Granger CV, Karmarkar AM, Graham JE, The Uniform Data System for Medical Rehabilitation: report of patients with traumatic spinal cord injury discharged from rehabilitation programs in 2002-2010. Am J Phys Med Rehabil. 2012;91(4):289-299. doi:10.1097/PHM.0b013e31824ad2fd 22407160 PMC3392040

[30] Granger CV, Markello SJ, Graham JE, Deutsch A, Reistetter TA, Ottenbacher KJ The Uniform Data System for Medical Rehabilitation: report of patients with traumatic brain injury discharged from rehabilitation programs in 2000-2007. Am J Phys Med Rehabil. 2010;89(4):265-278. doi:10.1097/PHM.0b013e3181d3eb20 20299850 PMC2918420

[31] Granger CV, Markello SJ, Graham JE, Deutsch A, Reistetter TA, Ottenbacher KJ The Uniform Data System for Medical Rehabilitation: report of patients with lower limb joint replacement discharged from rehabilitation programs in 2000-2007. Am J Phys Med Rehabil. 2010;89(10):781-794. doi:10.1097/PHM.0b013e3181f1c83a 20855979 PMC3132401

[32] Leland NE, Gozalo P, Christian TJ, An examination of the first 30 days after patients are discharged to the community from hip fracture postacute care. Med Care. 2015;53(10):879-887. doi:10.1097/MLR.0000000000000419 26340664 PMC4570868

[33] Cary MP Jr, Prvu Bettger J, Jarvis JM, Ottenbacher KJ, Graham JE Successful community discharge following postacute rehabilitation for Medicare beneficiaries: analysis of a patient-centered quality measure. Health Serv Res. 2018;53(4):2470-2482. doi:10.1111/1475-6773.12796 29134630 PMC6052014

[34] Department of Health and Human Services Medicare Program; Advancing Care Coordination Through Episode Payment Models (EPMs); Cardiac Rehabilitation Incentive Payment Model; and Changes to the Comprehensive Care for Joint Replacement Model (CJR); Proposed Rule. *Fed Regist* https://www.gpo.gov/fdsys/pkg/FR-2016-08-02/pdf/2016-17733.pdf. August 2, 2016. Accessed July 4, 2018. 28071874

