# Supplementary material for: Facility and Geographic Variation in Rates of Successful Community Discharge After Inpatient Rehabilitation Among Medicare Fee-for-Service Beneficiaries
Source: JAMA Netw Open. 2018 Nov 9;1(7):e184332. doi: 10.1001/jamanetworkopen.2018.4332 (PMC6324386; doi:10.1001/jamanetworkopen.2018.4332)
Supplement: Supplement. — eTable 1. Hierarchical Logistic Regression Model Estimating Successful Discharge to the Community After Inpatient Rehabilitation eTable 2. Risk-Standardized State Rates of Successful Community Discharge After Inpatient Rehabilitation and Corresponding 95% Confidence Intervals [file jamanetwopen-1-e184332-s001.pdf]

Supplementary Online Content

Middleton A, Graham JE, Prvu Bettger J, Haas A, Ottenbacher KJ. Facility and geographic variation in rates of successful community discharge after inpatient rehabilitation among Medicare fee-for-service beneficiaries. *JAMA Netw Open*. 2018;1(7):e184332. doi:10.1001/jamanetworkopen.2018.4332

eTable 1. Hierarchical logistic regression model estimating successful discharge to the community following inpatient rehabilitation
eTable 2. Risk standardized state rates of successful community discharge following inpatient rehabilitation and corresponding 95% confidence intervals

This supplementary material has been provided by the authors to give readers additional information about their work.

© 2018 Middleton A et al. *JAMA Network Open.*

eTable 1. Hierarchical logistic regression model estimating successful discharge to the community following inpatient rehabilitation

Description: The table below presents the parameter estimates from the hierarchical logistic regression model estimating successful discharge to the community following inpatient rehabilitation. We replicated as closely as possible the risk-adjustors specified for the Discharge to Community – Post-Acute Care Inpatient Rehabilitation Facility Quality Reporting Program measure.[a] The cohort included 487,862 Medicare fee-for-service beneficiaries discharged from inpatient rehabilitation between 12/31/13 and 10/01/15.

Patient Characteristic	Odds Ratio	95% CI	
		Lower	Upper
**Sex, age category**			
Female, 18-64	Reference		
Female, 66-74	0.91	0.87	0.94
Female, 75-79	0.78	0.75	0.81
Female, 80-84	0.66	0.64	0.69
Female, 85-89	0.54	0.52	0.56
Female, 90-94	0.48	0.46	0.50
Female, 95+	0.42	0.39	0.45
Male, 18-64	0.96	0.93	1.00
Male, 65-74	0.90	0.87	0.93
Male, 75-79	0.79	0.76	0.82
Male, 80-84	0.70	0.67	0.73
Male, 85-89	0.57	0.55	0.59
Male, 90-94	0.49	0.47	0.52
Male, 95+	0.42	0.38	0.46
**Original reason for Medicare entitlement**			
Age	Reference		
Disability or end-stage renal disease	0.92	0.90	0.94
**Principal diagnosis from index hospitalization** (CCS groupings)			
Osteoarthritis (203) and Complications of pregnancy, childbirth, and the puerperium (179-196)	Reference		
Infectious and parasitic diseases: Septicemia (2)	0.73	0.69	0.79
Infectious and parasitic diseases: Other (1, 3-10)	0.71	0.62	0.81
Neoplasms (11-15, 18, 20, 26, 28-34, 36-41, 43-47)	0.60	0.54	0.67
Endocrine disorders (48, 51, 53, 54)	0.67	0.59	0.76
Diabetes with and without complications (49, 50)	0.71	0.65	0.77
Nutritional deficiency and other nutritional disorders (52, 58)	0.59	0.51	0.69
Fluid/electrolyte disorders (55)	0.63	0.57	0.69
Diseases of blood and blood-forming organs (56-57, 59-64)	0.60	0.54	0.67
Diseases of the nervous system: Meningitis, encephalitis, other CNS infection (76-78)	0.49	0.43	0.56
Diseases of the nervous system: Parkinson's disease; multiple sclerosis; other hereditary and degenerative nervous system disease; paralysis (79-82)	0.64	0.59	0.70
Diseases of the nervous system: Epilepsy; convulsions (83)	0.72	0.65	0.79
Diseases of the nervous system: Other nervous system disorders (95)	0.66	0.61	0.72
Diseases of the circulatory system: Heart valve disorders (96)	0.70	0.64	0.77
Diseases of the circulatory system: Carditis; other heart disease (97, 104)	0.62	0.53	0.73
Diseases of the circulatory system: Essential hypertension; hypertension with complications and secondary hypertension (98, 99)	0.72	0.65	0.79

© 2018 Middleton A et al. *JAMA Network Open.*

eTable 1 (cont.)

**Principal diagnosis from index hospitalization** (CCS groupings)	**Odds Ratio**	**95% CI**	
		**Lower**	**Upper**
Diseases of the circulatory system: Acute myocardial infarction; cardiac arrest (100, 107)	0.67	0.61	0.73
Diseases of the circulatory system: Coronary atherosclerosis and other heart disease; nonspecific chest pain (101, 102)	0.71	0.65	0.78
Diseases of the circulatory system: Pulmonary heart disease (103)	0.83	0.74	0.93
Diseases of the circulatory system: Conduction disorders; cardiac dysrhythmias (105, 106)	0.73	0.68	0.79
Diseases of the circulatory system: Non-hypertensive congestive heart failure (108)	0.62	0.57	0.66
Diseases of the circulatory system: Acute cerebrovascular disease; occlusion or stenosis of precerebral arteries; other cerebrovascular disease; late effects of cerebrovascular disease (109-111, 113)	0.59	0.55	0.63
Diseases of the circulatory system: Transient cerebral ischemia (112)	0.85	0.76	0.95
Diseases of the circulatory system: Peripheral and visceral atherosclerosis (114)	0.64	0.58	0.70
Diseases of the circulatory system: Aortic, peripheral, and visceral artery aneurysms (115)	0.70	0.62	0.79
Diseases of the circulatory system: Aortic and peripheral arterial embolism or thrombosis; other circulatory disease (116, 117)	0.65	0.59	0.71
Diseases of the circulatory system: Phlebitis; varicose veins; hemorrhoids; other diseases of veins and lymphatics (118-121)	0.65	0.58	0.73
Diseases of the respiratory system: Pneumonia; influenza; acute bronchitis; other upper respiratory infections (122, 123, 125-126)	0.81	0.75	0.87
Diseases of the respiratory system: Acute and chronic tonsillitis; pleurisy, pneumothorax, or pulmonary collapse; lung disease due to external agents; other lower or upper respiratory disease (124, 130, 132-134)	0.68	0.61	0.76
Diseases of the respiratory system: Chronic obstructive pulmonary disease; asthma (127, 128)	0.62	0.57	0.67
Diseases of the respiratory system: Aspiration pneumonitis (129)	0.68	0.61	0.76
Diseases of the respiratory system: Adult respiratory failure (131)	0.69	0.64	0.75
Diseases of the digestive system: Intestinal infection; disorders of teeth and jaw; disease of mouth excluding dental; esophageal disorders; gastroduodenal ulcer; gastritis and duodenitis; other disorders of stomach and duodenum; appendicitis; abdominal hernia; regional enteritis and ulcerative colitis; diverticulosis; anal and rectal conditions; peritonitis and intestinal abscess; noninfectious gastroenteritis; other gastrointestinal disorders (135-144, 146-148, 154, 155)	0.70	0.65	0.76
Diseases of the digestive system: Intestinal obstruction without hernia (145)	0.85	0.77	0.95
Diseases of the digestive system: Biliary tract disease; alcohol-related liver disease; other liver diseases; pancreatic disorders (149-152)	0.74	0.67	0.82
Diseases of the digestive system: Gastrointestinal hemorrhage (153)	0.74	0.67	0.82
Diseases of the genitourinary system: Nephritis, nephrosis, or renal sclerosis; calculus of urinary tract; other disease of kidney and ureters; other diseases of bladder and urethra; genitourinary symptoms; hyperplasia of prostate; inflammatory conditions of male genital organs; other male genital disorders; inflammatory diseases of female pelvic organs; endometriosis; prolapse of female genital organs; menstrual disorders; ovarian cyst; menopausal disorders; female infertility; other female genital disorders (156, 160-166, 168-175)	0.63	0.55	0.72
Diseases of the genitourinary system: Acute renal failure; chronic kidney disease (157,158)	0.69	0.64	0.74
Diseases of the genitourinary system: Urinary tract infections (159)	0.67	0.62	0.73
Diseases of the skin and subcutaneous tissue: Nonmalignant breast conditions; skin and subcutaneous tissue infections; other inflammatory condition of skin; chronic ulcer of skin; other skin disorders (167, 197-200)	0.76	0.69	0.83

© 2018 Middleton A et al. *JAMA Network Open.*

eTable 1 (cont.)

**Principal diagnosis from index hospitalization** (CCS groupings)	**Odds Ratio**	**95% CI**	
		**Lower**	**Upper**
Diseases of the musculoskeletal system: Infective arthritis and osteomyelitis (201)	0.61	0.53	0.70
Diseases of the musculoskeletal system: Rheumatoid arthritis, systematic lupus erythematosus and connective tissue disorders; other connective tissue disease (202, 210, 211)	0.70	0.64	0.76
Diseases of the musculoskeletal system: Other non-traumatic joint disorders; osteoporosis (204, 206)	0.79	0.68	0.92
Diseases of the musculoskeletal system: Spondylosis, intervertebral disc disorders, and other back problems (205)	0.86	0.80	0.92
Diseases of the musculoskeletal system: Pathological fracture (207)	0.62	0.57	0.68
Diseases of the musculoskeletal system: Acquired foot deformities; other acquired deformities (208, 209)	0.89	0.80	0.99
Diseases of the musculoskeletal system: Other bone disease and musculoskeletal deformities (212)	0.74	0.67	0.82
Congenital anomalies: Cardiac and circulatory; digestive; genitourinary; nervous system; other (213-217)	0.84	0.72	0.98
Injury and poisoning: Trauma-related joint disorders and dislocations (225)	0.57	0.46	0.69
Injury and poisoning: Fracture of neck of femur (226)	0.61	0.56	0.65
Injury and poisoning: Spinal cord injury (227)	0.48	0.41	0.55
Injury and poisoning: Skull and face fractures; other fractures (228, 231)	0.71	0.66	0.77
Injury and poisoning: Fracture of upper limb (229)	0.55	0.50	0.60
Injury and poisoning: Fracture of lower limb (230)	0.50	0.46	0.54
Injury and poisoning: Sprains and strains; superficial injury/contusion (232, 239)	0.73	0.65	0.82
Injury and poisoning: Intracranial injury (233)	0.49	0.45	0.53
Injury and poisoning: Crushing injury or internal injury (234)	0.87	0.76	1.00
Injury and poisoning: Open wounds of head, neck, or trunk; open wounds of extremities; burns; other injuries and conditions due to external causes (235, 236, 240, 244)	0.67	0.59	0.77
Injury and poisoning: Complications of device, implant, or graft; complications of surgical procedures or medical care (237-238)	0.65	0.61	0.69
Injury and poisoning: Poisoning by psychotropic agents, poisoning by other medications and drugs; poisoning by nonmedicinal substances (241-243)	0.78	0.64	0.94
Other conditions: Syncope; fever of unknown origin; lymphadenitis; shock; nausea and vomiting; abdominal pain; malaise and fatigue; allergic reactions; rehabilitation care, fitting of prostheses, or adjustment of devices; administrative/social admission; medical examinations/evaluation; other aftercare; other screening for suspected conditions (245-247, 249-259)	0.75	0.69	0.82
Other conditions: Gangrene (248)	0.64	0.57	0.71
Other conditions: Mental illness (650-670)	0.75	0.66	0.85
**Inpatient Rehabilitation Facility Case-Mix Groups**			
Stroke: Motor score > 44.45 (CMGs: 0101-0103)	Reference		
Stroke: Motor score 26.15-44.45 (CMGs: 0104-0107)	0.54	0.51	0.57
Stroke: Motor score <26.15 and age>84.5; motor score 22.35-26.15 and age<84.5 (CMGs: 0108-0109)	0.21	0.20	0.23
Stroke: Motor score <22.35 and age <84.5 (CMG: 0110)	0.13	0.12	0.14
Traumatic Brain Injury: Motor score >28.75 (CMGs: 0201-0205)	0.52	0.48	0.57
Traumatic Brain Injury: Motor score <28.75 (CMGs: 0206-0207)	0.22	0.20	0.24
Non-traumatic Brain Injury: Motor score >35.05 (CMGs: 0301-0302)	0.55	0.50	0.59
Non-traumatic Brain Injury: Motor score <35.05 (CMGs: 0303-0304)	0.25	0.23	0.27

© 2018 Middleton A et al. *JAMA Network Open.*

eTable 1 (cont.)

Inpatient Rehabilitation Facility Case-Mix Groups	Odds Ratio	95% CI	
		Lower	Upper
Traumatic Spinal Cord Injury: All (CMGs: 0401-0405)	0.26	0.23	0.29
Non-traumatic Spinal Cord Injury: Motor score >31.25 (CMGs: 0501-0503)	0.55	0.50	0.60
Non-traumatic Spinal Cord Injury: Motor score <31.25 (CMGs: 0504-0506)	0.19	0.17	0.20
Neurological: Motor score >37.35 (CMGs: 0601-0602)	0.53	0.49	0.57
Neurological: Motor score <37.35 (CMGs: 0603-0604)	0.26	0.25	0.28
Fracture of Lower Extremity: Motor score >28.15 (CMGs: 0701-0703)	0.68	0.63	0.74
Fracture of Lower Extremity: Motor score <28.15 (CMG: 0704)	0.25	0.23	0.27
Replacement of Lower Extremity Joint: Motor score >28.65 (CMGs: 0801-0804)	0.69	0.63	0.75
Replacement of Lower Extremity Joint: Motor score <28.65 (CMGs: 0805-0806)	0.28	0.26	0.30
Other Orthopedic: Motor score >24.15 (CMGs: 0901-0903)	0.43	0.40	0.46
Other Orthopedic: Motor score <24.15 (CMG: 0904)	0.17	0.16	0.19
Amputation, Lower Extremity: Motor score >36.25 (CMGs:1001-1002)	0.51	0.45	0.57
Amputation, Lower Extremity: Motor score <36.25 (CMG:1003) & Amputation, Non-Lower Extremity (CMGs: 1101-1102)	0.26	0.24	0.29
Osteoarthritis: All (CMGs: 1201-1203)	0.31	0.27	0.36
Rheumatoid, Other Arthritis: All (CMGs: 1301-1303)	0.33	0.29	0.38
Cardiac: Motor score >38.55 (CMGs: 1401-1402)	0.57	0.53	0.62
Cardiac: Motor score <38.55 (CMGs: 1403-1404)	0.33	0.31	0.36
Pulmonary: Motor score >39.05 (CMGs: 1501-1502)	0.48	0.43	0.53
Pulmonary: Motor score <39.05 (CMGs: 1503-1504)	0.29	0.27	0.31
Pain Syndrome: All (CMGs: 1601-1603)	0.33	0.29	0.37
Major Multiple Trauma Without Brain or Spinal Cord Injury (CMGs: 1701-1704)	0.29	0.27	0.31
Major Multiple Trauma With Brain or Spinal Cord Injury (CMGs: 1801-1803)	0.29	0.26	0.33
Guillain Barre (CMGs: 1901-1903)	0.26	0.22	0.31
Miscellaneous (CMGs: 2001-2004), Burns (CMG 2101), Short-stay cases (CMG: 5001)	0.30	0.28	0.32
**Surgical categories based on procedures during prior hospitalization** (reference = “No” for each category)			
Cardio Thoracic	1.301	1.24	1.38
Obstetrics/Gynecology or Urology	1.14	1.02	1.28
Neurosurgery	1.16	1.10	1.21
Orthopedics	1.12	1.08	1.15
General	1.24	1.19	1.30
Vascular	1.27	1.20	1.33
**Dialysis during prior hospitalization without ESRD diagnosis** (reference= “No”)	1.04	0.99	1.10
**Length of stay for prior hospitalization or indicator for prior stay in psychiatric hospital**			
1-3 days	Reference		
Psychiatric hospital	0.73	0.53	1.02
4-5 days	0.89	0.87	0.91
6-8 days	0.77	0.76	0.78
9-10 days	0.71	0.69	0.73
11-14 days	0.65	0.63	0.67
15-20 days	0.60	0.58	0.62
21-30 days	0.57	0.55	0.60
31+ days	0.49	0.46	0.53

© 2018 Middleton A et al. *JAMA Network Open.*

eTable 1 (cont.)

	Odds Ratio	95% CI	
		Lower	Upper
**Comorbidities** (HCCs; reference=“No” for each comorbidity)[b]			
Opportunistic infections	0.90	0.83	0.96
Metastatic cancer and acute leukemia	0.71	0.67	0.74
Lung and other severe cancers	0.77	0.73	0.80
Lymphoma and other cancers	0.87	0.83	0.90
Colorectal, bladder, and other cancers	0.96	0.91	1.01
Breast, prostate, and other cancers and tumors	0.92	0.89	0.95
Diabetes with acute complications	0.93	0.87	1.00
Diabetes with chronic complications; diabetes without complication	0.94	0.93	0.96
Protein-calorie malnutrition	0.82	0.81	0.84
Morbid obesity	0.96	0.94	0.98
Other significant endocrine and metabolic disorders	0.901	0.89	0.93
End-stage liver disease	0.77	0.71	0.83
Cirrhosis of liver	0.79	0.74	0.84
Chronic hepatitis	0.85	0.71	1.01
Bone/joint/muscle infections/necrosis[a]	0.95	0.91	0.99
Rheumatoid arthritis and inflammatory connective tissue disease	0.95	0.93	0.97
Severe hematological disorders	0.90	0.85	0.95
Disorders of immunity	0.95	0.91	0.99
Coagulation defects and other specified hematological disorders[a]	0.96	0.93	0.98
Dementia with complications; dementia without complication	0.80	0.79	0.82
Schizophrenia	0.81	0.75	0.87
Major depressive, bipolar, and paranoid disorders	0.84	0.82	0.87
Quadriplegia	0.82	0.78	0.87
Paraplegia	0.85	0.81	0.90
Spinal cord disorders/injuries	0.89	0.85	0.93
Amyotrophic lateral sclerosis and other motor neuron disease[a]	0.87	0.69	1.09
Cerebral palsy	0.87	0.77	0.98
Muscular dystrophy; multiple sclerosis	0.96	0.91	1.02
Parkinson's and Huntington's diseases	0.92	0.90	0.95
Coma, brain compression/anoxic damage	0.87	0.84	0.91
Respirator dependence/tracheostomy status	0.93	0.87	0.99
Congestive heart failure	0.87	0.85	0.88
Acute myocardial infarction[a]	0.94	0.90	0.99
Unstable angina and other acute ischemic heart disease[a]	0.95	0.90	1.00
Specified heart arrhythmias	0.88	0.87	0.89
Cerebral hemorrhage	0.82	0.79	0.85
Ischemic or unspecified stroke	0.83	0.81	0.86
Hemiplegia/hemiparesis	0.90	0.88	0.92
Monoplegia, other paralytic syndromes	0.99	0.94	1.05
Atherosclerosis of the extremities with ulceration or gangrene[a]	0.81	0.76	0.86

© 2018 Middleton A et al. *JAMA Network Open.*

eTable 1 (cont.)

**Comorbidities** (HCCs; reference="No" for each comorbidity)[a]	**Odds Ratio**	**95% CI**	
		**Lower**	**Upper**
Vascular disease with complications[a]	0.97	0.92	1.01
Vascular disease[a]	0.93	0.92	0.95
Cystic fibrosis; chronic obstructive pulmonary disease; fibrosis of lung and other chronic lung disorders	0.89	0.88	0.91
Aspiration and specified bacterial pneumonias[a]	0.94	0.90	0.97
Dialysis status	0.85	0.79	0.91
Acute renal failure; unspecified renal failure	0.85	0.84	0.87
Chronic kidney disease (Stage 5)	0.81	0.76	0.86
Chronic kidney disease, severe (Stage 4)	0.88	0.85	0.90
Pressure ulcer of skin with necrosis through to muscle, tendon, or bone; pressure ulcer of skin with full thickness skin loss[a]	0.95	0.87	1.04
Pressure ulcer of skin with partial thickness skin loss[a]	0.97	0.89	1.06
Pressure pre-ulcer skin changes or unspecified stage[a]	0.79	0.75	0.84
Chronic ulcer of skin, except pressure	0.88	0.85	0.91
Vertebral fractures without spinal cord injury	0.89	0.86	0.93
Hip fracture/dislocation[a]	0.80	0.76	0.84
Complications of specified implanted device or graft[a]	0.87	0.83	0.91
Artificial openings for feeding or elimination[a]	0.85	0.81	0.90
Amputation status, lower limb/amputation complications; amputation status, upper limb	0.92	0.88	0.96
**Number of acute care stays over year prior**			
0	Reference		
1	0.91	0.90	0.93
2	0.85	0.83	0.87
3	0.78	0.76	0.80
4	0.73	0.70	0.76
5	0.67	0.63	0.71
6	0.66	0.61	0.71
7	0.55	0.50	0.61
8	0.46	0.40	0.53
9	0.38	0.31	0.47
10+	0.30	0.26	0.36

Abbreviations: CI, confidence interval; CCS, Clinical Classifications Software; CMG, case-mix group; ESRD, end stage renal disease; HCC, Hierarchical Condition Categories

a. RTI International. Measure Specifications for Measures Adopted in the FY 2017 IRF QRP Final Rule. https://www.cms.gov/Medicare/Quality-Initiatives-Patient-Assessment-Instruments/IRF-Quality-Reporting/Downloads/Measure-Specifications-for-FY17-IRF-QRP-Final-Rule.pdf. Accessed July 4, 2018.

b. Indicates the HCC identified using diagnosis codes from only prior hospitalization. All other HCCs identified using diagnosis codes from all acute stays over prior year.

© 2018 Middleton A et al. *JAMA Network Open.*

eTable 2. Risk standardized state rates of successful community discharge following inpatient rehabilitation and corresponding 95% confidence intervals

Description: The table below presents risk-standardized state rates of successful discharge to the community following inpatient rehabilitation. We replicated as closely as possible the risk-adjustors specified for the Discharge to Community – Post-Acute Care Inpatient Rehabilitation Facility Quality Reporting Program measure.[a] The cohort included 487,862 Medicare fee-for-service beneficiaries discharged from inpatient rehabilitation between 12/31/13 and 10/01/15.

State	Risk Standardized State Rate (95% CI)
Alabama	67.3 (65.1-69.1)
Alaska	70.0 (65.5-73.5)
Arizona	66.6 (64.7-68.4)
Arkansas	63.5 (61.7-65.3)
California	68.2 (66.4-70.0)
Colorado	65.3 (63.6-67.1)
Connecticut	60.0 (57.8-62.4)
Delaware	62.6 (59.9-64.9)
Florida	64.7 (62.5-66.1)
Georgia	66.2 (64.1-67.8)
Hawaii	73.3 (70.5-75.7)
Idaho	67.1 (64.5-69.7)
Illinois	59.3 (57.2-60.9)
Indiana	60.9 (59.0-62.7)
Iowa	61.1 (58.7-63.3)
Kansas	62.3 (60.4-64.2)
Kentucky	63.1 (61.2-64.9)
Louisiana	61.3 (59.5-63.2)
Maine	58.5 (56.1-61.2)
Maryland	65.6 (63.3-67.4)
Massachusetts	55.9 (53.9-57.8)
Michigan	61.7 (59.7-63.5)
Minnesota	62.8 (60.9-64.9)
Mississippi	63.6 (61.5-65.6)
Missouri	60.8 (58.8-62.6)
Montana	63.9 (61.1-66.7)
Nebraska	58.9 (56.5-61.3)
Nevada	59.9 (58.0-61.8)
New Hampshire	57.0 (54.9-59.3)
New Jersey	57.7 (55.7-59.3)
New Mexico	64.0 (61.7-66.1)
New York	59.2 (57.3-60.6)
North Carolina	65.1 (63.3-67.0)
North Dakota	59.3 (56.9-62.3)
Ohio	61.0 (59.2-62.6)
Oklahoma	62.7 (60.8-64.4)
Oregon	70.3 (67.6-72.5)
Pennsylvania	64.1 (62.5-65.8)
Rhode Island	60.2 (57.2-63.2)
South Carolina	68.1 (66.2-70.0)
South Dakota	64.2 (60.9-67.1)
Tennessee	66.5 (64.6-68.3)
Texas	63.6 (61.9-65.3)

© 2018 Middleton A et al. *JAMA Network Open.*

Utah	61.9 (59.5-64.4)
Vermont	60.6 (57.1-64.1)
Virginia	65.0 (63.0-66.8)
Washington	66.3 (64.4-68.1)
West Virginia	66.5 (64.1-68.5)
Wisconsin	63.6 (61.5-65.6)
Wyoming	63.4 (60.9-65.8)

Abbreviations: CI, confidence interval

a. RTI International. Measure Specifications for Measures Adopted in the FY 2017 IRF QRP Final Rule. https://www.cms.gov/Medicare/Quality-Initiatives-Patient-Assessment-Instruments/IRF-Quality-Reporting/Downloads/Measure-Specifications-for-FY17-IRF-QRP-Final-Rule.pdf. Accessed July 4, 2018.

© 2018 Middleton A et al. *JAMA Network Open.*
